# Growth and mortality of aerobic anoxygenic phototrophs in the North Pacific Subtropical Gyre

**DOI:** 10.1128/aem.00032-24

**Published:** 2024-03-29

**Authors:** Michal Koblížek, Isabel Ferrera, Eva Kolářová, Solange Duhamel, Kimberly J. Popendorf, Josep M. Gasol, Benjamin A. S. Van Mooy

**Affiliations:** 1 Laboratory of Anoxygenic Phototrophs, Institute of Microbiology, Czech Academy of Science, Třeboň, Czechia; 2 Centro Oceanográfico de Málaga, Instituto Español de Oceanografía (IEO-CSIC), Fuengirola, Málaga, Spain; 3 Department of Cellular and Molecular Biology, University of Arizona, Tucson, Arizona, USA; 4 Rosenstiel School of Marine, Atmospheric, and Earth Science, University of Miami, Coral Gables, Florida, USA; 5 Institut de Ciències del Mar (ICM-CSIC), Barcelona, Catalonia, Spain; 6 Department of Marine Chemistry and Geochemistry, Woods Hole Oceanographic Institution, Woods Hole, Massachusetts, USA; University of Delaware, Lewes, Delaware, USA

**Keywords:** aerobic anoxygenic phototrophs, bacteriochlorophyll *a*, marine bacteria, North Pacific Subtropical Gyre, Station ALOHA

## Abstract

Aerobic anoxygenic phototrophic (AAP) bacteria harvest light energy using bacteriochlorophyll-containing reaction centers to supplement their mostly heterotrophic metabolism. While their abundance and growth have been intensively studied in coastal environments, much less is known about their activity in oligotrophic open ocean regions. Therefore, we combined *in situ* sampling in the North Pacific Subtropical Gyre, north of O'ahu island, Hawaii, with two manipulation experiments. Infra-red epifluorescence microscopy documented that AAP bacteria represented approximately 2% of total bacteria in the euphotic zone with the maximum abundance in the upper 50 m. They conducted active photosynthetic electron transport with maximum rates up to 50 electrons per reaction center per second. The *in situ* decline of bacteriochlorophyll concentration over the daylight period, an estimate of loss rates due to predation, indicated that the AAP bacteria in the upper 50 m of the water column turned over at rates of 0.75–0.90 d^−1^. This corresponded well with the specific growth rate determined in dilution experiments where AAP bacteria grew at a rate 1.05 ± 0.09 d^−1^. An amendment of inorganic nitrogen to obtain N:P = 32 resulted in a more than 10 times increase in AAP abundance over 6 days. The presented data document that AAP bacteria are an active part of the bacterioplankton community in the oligotrophic North Pacific Subtropical Gyre and that their growth was mostly controlled by nitrogen availability and grazing pressure.

**IMPORTANCE:**

Marine bacteria represent a complex assembly of species with different physiology, metabolism, and substrate preferences. We focus on a specific functional group of marine bacteria called aerobic anoxygenic phototrophs. These photoheterotrophic organisms require organic carbon substrates for growth, but they can also supplement their metabolic needs with light energy captured by bacteriochlorophyll. These bacteria have been intensively studied in coastal regions, but rather less is known about their distribution, growth, and mortality in the oligotrophic open ocean. Therefore, we conducted a suite of measurements in the North Pacific Subtropical Gyre to determine the distribution of these organisms in the water column and their growth and mortality rates. A nutrient amendment experiment showed that aerobic anoxygenic phototrophs were limited by inorganic nitrogen. Despite this, they grew more rapidly than average heterotrophic bacteria, but their growth was balanced by intense grazing pressure.

## INTRODUCTION

Aerobic anoxygenic phototrophic (AAP) bacteria are facultative photoheterotrophic organisms that harvest light energy using bacteriochlorophyll (BChl)-containing photosynthetic complexes (1, 2). AAP bacteria are a common part of the microbial community in the upper ocean (3), representing ~1%–10% of the total bacteria in the euphotic zone (4–8). Analyses of the *pufM* gene diversity documented that marine AAP bacteria represent a complex community composed of various classes of Alpha- and Gammaproteobacteria (9–17).

Since AAP bacteria do not fix inorganic carbon, they require organic substrates for growth. Laboratory experiments demonstrated that the ability to utilize light provides an auxiliary source of energy for their cellular metabolism (18, 19). When illuminated, AAP bacteria increase their bacterial growth efficiency and produce more biomass from a given amount of organic substrate (20–22). However, it turned out to be more difficult to establish the contribution of AAP metabolism under natural field conditions (23). Light exposure enhanced the growth of AAP bacteria in bottle incubations, but the stimulatory effect was relatively weak (24, 25). Dilution and grazer removal experiments performed in marine coastal waters showed that AAP bacteria grow faster than average heterotrophic bacteria, but that faster growth is balanced by intense grazing (26, 27).

While most of the information about the activity of AAP bacteria originates from coastal regions, much less is known from the open ocean. This greatly limits our understanding of the role of AAP bacteria in global marine microbial food webs. To contribute knowledge on AAP ecology in the open ocean, we conducted a sampling campaign in the North Pacific Subtropical Gyre (NPSG) in the vicinity of A Long-term Oligotrophic Habitat Assessment (ALOHA) Station, north of O'ahu island, Hawaii (28, 29). The presence of AAP bacteria within the euphotic zone at Station ALOHA was first documented by Cottrell et al. (5) using infra-red (IR) epifluorescence microscopy. A metatranscriptomic study of microbial activity at that site reported that *pufA* and *pufB* genes encoding light-harvesting antenna of AAP bacteria were, despite their relatively low abundance, one the most intensively expressed genes in the water column (30). In contrast, a qPCR study conducted in the waters off-shore of O'ahu reported low copy numbers of *pufM* genes representing less than 1% of total bacteria (13). Facing these contrasting results, we used infra-red epifluorescence microscopy to enumerate AAP abundance within the euphotic zone near Station ALOHA. To determine bottom-up limitations of their growth, we conducted an on-deck nutrient amendment experiment. The specific growth rate of AAP bacteria was determined in a dilution experiment using microscopy counts, and finally, the top-down pressure was estimated from diel changes of BChl concentration in the water column. This work contributes to establish the role of AAP bacteria in marine microbial food webs and elucidate their contribution to biogeochemical cycling in the upper open ocean.

## RESULTS AND DISCUSSION

### Distribution and activity of AAP bacteria in the water column

**Fig 1:**
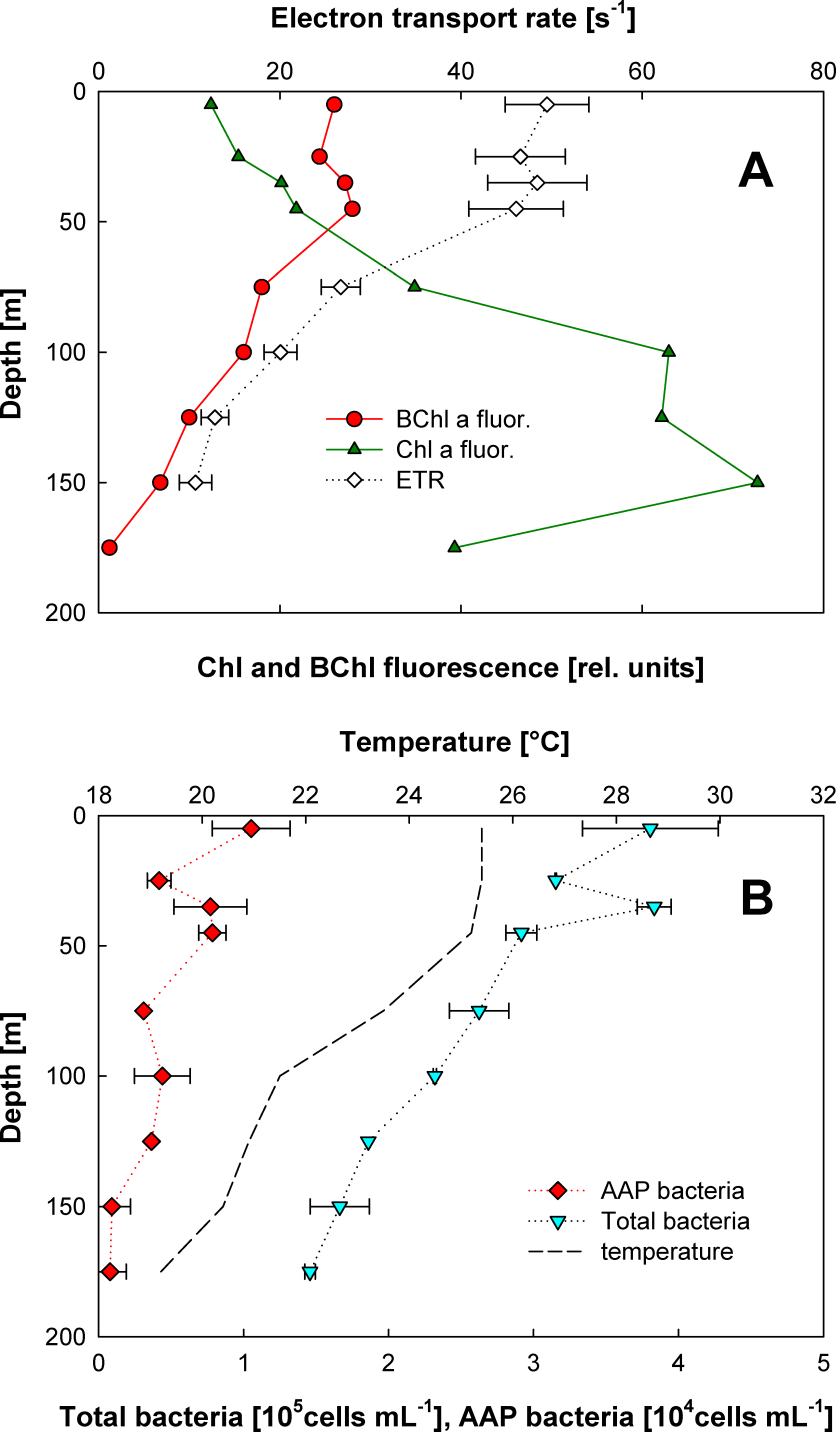
Distribution of the core parameters in the euphotic zone of the NPSG, cast #27, 18 July 2010. (**A)** BChl *a* and Chl *a* fluorescence signal and electron transport rate through the bacterial reaction centers (RCs). (**B)** Distribution of AAP and total prokaryote abundance were determined by epifluorescence microscopy (see Material and Methods).

The depth profile of BChl *a* and chlorophyll (Chl) *a* signals in the upper 250 m was recorded using IR fluorometry during seven casts spaced over 10 days of the cruise. The BChl *a* signals were observed consistently in all the samples collected in the upper 175 m. Figure 1A shows a typical depth distribution of the fluorescence signals recorded during cast #27. The highest signals corresponding to ~1 ng BChl *a* L^−1^ were found in the mixed layer (upper 45 m). In contrast, the Chl *a* signal peaked at depths below 100 m. AAP bacteria were also enumerated by IR epifluorescence microscopy. They ranged between 700 and 10,000 cells mL^−1^, which represented 2.0 ± 0.7% of the total bacteria (Fig. 1B).

Our AAP counts from the NPSG are comparable with the 500 to 25,000 cells mL^−1^ reported by Cottrell et al. (5) in waters around O'ahu island. Similar numbers were reported also from oligotrophic regions such as the Sargasso Sea or the eastern Mediterranean Sea (6, 31–34). In the current study, we show that AAP bacteria were more abundant in the upper 50 m of the water column, while the Chl *a* maximum was below 100 m. Similar vertical separation of the BChl *a* and Chl *a* signals has also been observed in oligotrophic provinces of the Mediterranean Sea (33, 35).

The fluorescence kinetic measurements also offer information about the activity of photochemical reactions. We showed in our previous work with AAP cultures that the relaxation kinetics of the fluorometric protocol can be used to obtain information about the electron transport activity of their photosynthetic reaction centers (RCs) (36, 37). Here, we used for the first time this approach in the field to analyze the electron transport activity of the natural AAP community. The kinetic measurements documented that AAP bacteria in the upper 50 m exhibit maximum electron transport rates from 40 to 50 electrons RC^−1^ s^−1^. Below the mixed layer, the maximum electron transfer rates decreased. This shows that natural populations of AAP bacteria in the NPSG were photosynthetically active, though the determined electron transport rates were somewhat lower than rates (80–300 electrons RC^−1^ s^−1^) observed in laboratory cultures of the marine AAP bacterium *Roseobacter litoralis* (37). Due to low BChl *a* fluorescence signals and high background of the chlorophyll signal, it was not technically possible to determine accurately the yield of primary photochemistry *F*_*V*_/*F*_*M*_. However, in contrast to algae, this parameter is relatively stable in AAP bacteria (3) with only 10% reduction in high light-grown cultures (22).

### Top-down control of AAP growth

In our previous work, we showed that the mortality of AAP bacteria can be monitored from the diel changes of BChl *a* concentration (31, 38). This approach is based on the fact that AAP bacteria produce BChl *a* only during the night as light fully represses its synthesis (39, 40). As there is no new BChl *a* synthesis during the day, the removal of BChl *a*-containing cells by grazing or viral attack leads to a total pigment decline. On the other hand, cell division (during the day) has no effect on the total BChl *a* concentration as the daughter cells simply split the BChl *a* content. Under the assumption that grazing and viral lysis are the only cause of BChl *a* removal (no photobleaching or pigment degradation), the BChl *a* decline can be analyzed mathematically to determine AAP mortality (38).

**Fig 2:**
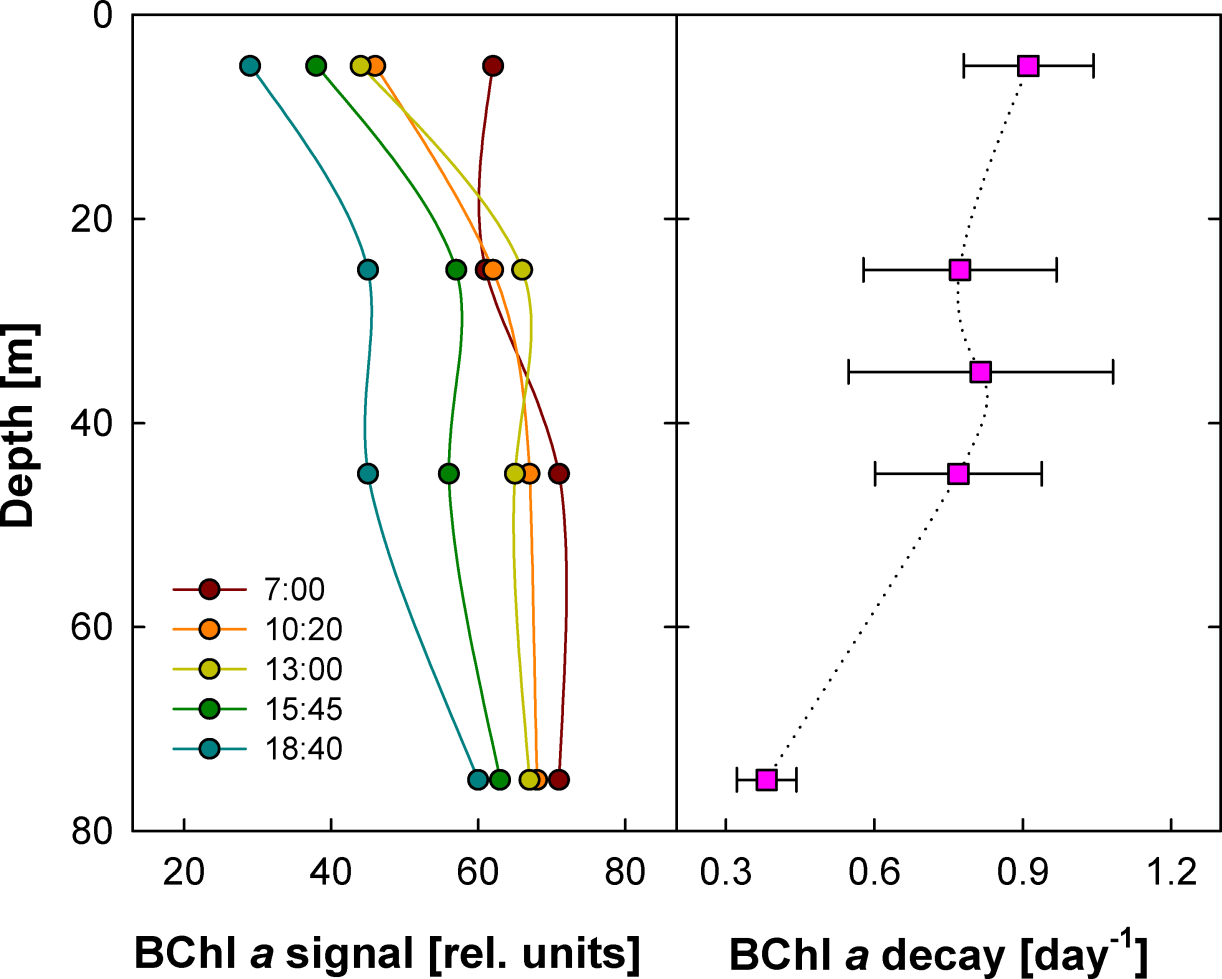
Left: Decline in BChl *a* fluorescence recorded at various depths on 15 July 2010. Colors indicate times of the individual casts (see legend). Right: Average BChl *a* decay rates calculated for each depth from two to four independent BChl *a* decay measurements performed on 14–17 July 2010 (error bars represent the standard deviations).

This approach has been used to determine AAP mortality rates in the surface waters of the Atlantic Ocean and the Mediterranean Sea (31, 33), but so far, no measurements have been performed in deeper parts of the water column. Here, we followed the changes in BChl *a* signals at 5, 25, 35, 45, and 75 m. During the 4 days of measurements, we observed a clear diel pattern. The highest signals were registered in the morning after sunrise, and they decreased during the day reaching their minima in the evening (Fig. 2). The BChl *a* decayed in the upper 50 m at rates between 0.75 and 0.9 d^−1^, with the highest rates at the surface. A lower value 0.45 d^−1^ was recorded at 75 m (Fig. 2).

As discussed previously, the BChl *a* decays can be used to estimate AAP mortality only under conditions in which there is no BChl *a* photobleaching. Anoxygenic photosystems seem to be relatively stable complexes, as photobleaching has never been observed in AAP laboratory cultures (38, 39). No indication of BChl *a* photobleaching was observed during the on-deck incubations of water samples in the Sargasso Sea (31). Likewise, we did not see any evidence of photobleaching in the presented measurements. In general, photobleaching is directly proportional to light intensity. Thus, it should be maximal at the surface and quickly vanish with depth. In contrast, our data show that BChl *a* decay rates were similar throughout the entire mixed zone (upper 50 m), ranging from 0.75 to 0.9 d^−1^. Therefore, we argue that the recorded BChl *a* decay rates can be used to assess the AAP mortality rates. The rates determined in the NPSG study are very similar to those determined using bottle incubations in the South Pacific (41) and South Atlantic Gyre (0.72–0.89 d^−1^) (31). Only slightly higher rates were found in the phosphorus-limited Sargasso Sea (0.91 and 1.03 d^−1^) (31).

**Fig 3:**
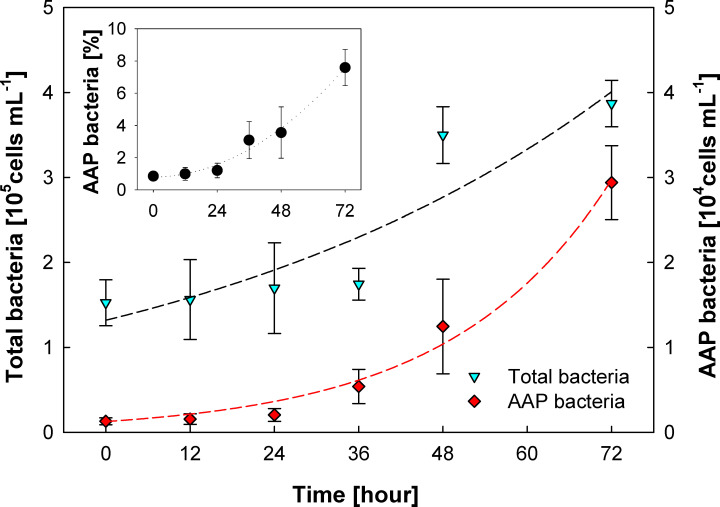
Dilution experiment. The seawater sample collected at 25-m depth was diluted 10-fold, and the regrowth of total prokaryotes [4′,6-diamidino-2-phenylindol (DAPI)] and AAP bacteria was followed for 3 days. The specific growth rate of total bacteria was *µ* = 0.37 ± 0.08 d^−1^, and for AAP bacteria, it was *µ* = 1.05 ± 0.09 d^−1^. The inset shows the relative increase in AAP bacteria expressed as percentage of the total DAPI numbers.

The results we obtained indicate that the AAP community in the NPSG was active and turned over rapidly. To further support this observation, we determined their specific growth rate using an on-deck dilution experiment. The collected seawater was 10× diluted with prefiltered seawater, and the growth of total and AAP bacteria was followed for 3 days by epifluorescence microscopy (see Material and Methods). The experiment showed that AAP bacteria grew at a rate of 1.05 ± 0.09 d^−1^ (Fig. 3). The growth rate of total bacteria was 0.37 ± 0.08 d^−1^, which is comparable to the growth rates usually found in oligotrophic waters using dilution experiments (42, 43), but lower than growth rates determined using radioactive substrates (41). Due to their faster growth, the proportion of AAP cells in the total bacterial pool increased from 1% to 8%. The specific growth rate we obtained corresponds relatively well to the AAP mortality rates estimated from the BChl *a* decay kinetics, which indicates that the AAP community in the NPSG was in dynamic equilibrium with growth, and mortality rates approximately balanced.

### Bottom-up control of AAP growth

Earlier studies from the phosphorus-limited Mediterranean Sea and the Adriatic Sea indicated that AAP bacteria may be more prone to phosphorus limitation than the average heterotrophic bacteria (24, 27). The eastern NPSG (including Station ALOHA) alternates between periods of phosphate sufficiency and limitation. Periods of phosphate limitation occur when the concentration of dissolved inorganic phosphate in surface waters drops below approximately 50–60 nmol L^−1^ (44). The concentration of inorganic phosphate at 25 m in our *T*_*0*_ experimental samples was 72 ± 3 nmol L^−1^ (see Table 1), and primary production was limited by nitrogen (45).

**TABLE 1:** Basic physicochemical and biological parameters of the water collected for the on-deck experiments^*a*^

Parameter	Value
Temperature	25.9°C
Depth	25 m
Chlorophyll	132 ± 17 ng L^−1^
Dissolved inorganic phosphorus	72 ± 3 nmol L^−1^
NH_4_^+^	25 ± 11 nmol L^−1^
NO_3_^−^	6.2 ± 2.0 nmol L^−1^
Heterotrophic bacteria	3.8 × 10^8^ *cells* L^−1^
*Prochlorococcus*	2.5 × 10^8^ *cells* L^−1^
*Synechococcus*	8.7 × 10^5^ *cells* L^−1^
Picoeukaryotes	7.6 × 10^5^ *cells* L^−1^
Leucine incorporation rate	895 ± 9 pmol L^−1^ d^−1^
Thymidine incorporation rate	57 ± 13 pmol L^−1^ d^−1^

^*a*^ Chemical data were taken from Popendorf et al. (2020) and Duhamel et al. (2014) (41, 45).

**Fig 4:**
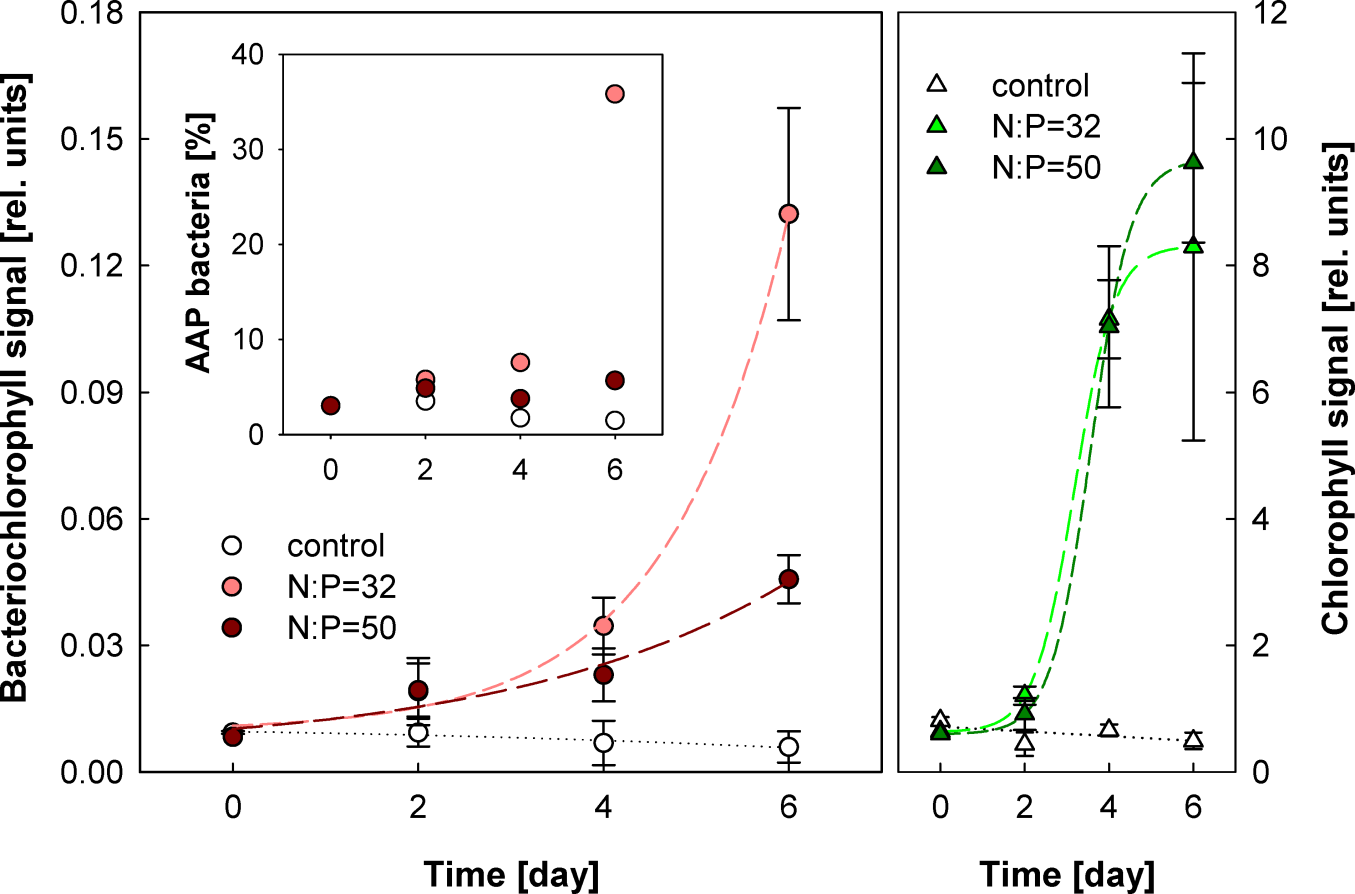
Nitrogen-enrichment experiment. The triplicate water samples were amended with an equimolar solution of NH_4_Cl and NaNO_3_ to obtain a nitrogen-to-phosphorus ratio = 32 or 50, and the changes in BChl *a* (circles) and Chl *a* fluorescence signals (triangles) were monitored by IR fluorometer every 2 days. The points report average values of the triplicates, and error bars indicate their standard deviations. The inset shows the percentage of AAP bacteria determined by epifluorescence microscopy.

To find out what the bottom-up limitation of AAP bacteria was, we performed a nitrogen amendment experiment where combined nitrogen (nitrate + ammonium) was added to elevate the N:P ratio to 32 or to 50, which corresponds to two or approximately three times the Redfield ratio (see Material and Methods). The addition of combined nitrogen induced approximately a 10-fold increase in the Chl *a* signal in both amendment treatments (Fig. 4). This is consistent with the increase in phytoplankton abundance and primary productivity reported by Duhamel et al. (45) from the same experiment. The nitrogen addition also stimulated the AAP community. After 2 days of incubation, the BChl *a* signal doubled in both nitrogen-amended treatments, while the control treatments remained approximately constant. In the later phase of the experiment, AAP bacteria continued growing. The growth in the later phase was more likely caused by input of new organic matter from phytoplankton than the nitrogen amendment directly. Interestingly, at the end of the experiment, BChl *a* signal was significantly higher in the N:P = 32 treatment (AAP abundance reached 1.68 × 10^5^ cells mL^−1^) than in the N:P = 50 treatment (Fig. 4). The reason for this effect is not clear, but we assume that it may be caused by a different phytoplankton and grazer community proliferating in N:P = 32 and N:P = 50 treatments. In fact, Duhamel et al. (45) found higher increase in picoalgae (including mixotrophic protists) abundance in the N:P = 50 treatment. These eukaryotes may have been responsible for removing part of the AAP cells in the N:P = 50 treatment. Total bacterial abundance also increased in response to the N:P = 32 amendment as reported by Duhamel et al. (45). Here, we found that this increase was mostly caused by AAP cells, which rose from 3% to approximately 36% of the total prokaryotes at the end of the experiment (Fig. 4 inset).

### Conclusions

While AAP bacteria have been studied in many marine habitats, including the open ocean, coastal seas, and estuaries (1), there is significantly less information about AAP distribution, growth, and mortality rates from nitrogen-limited oligotrophic gyres, which represent large parts of the global ocean (46). We found that, due to intense grazing pressure and nitrogen limitation, AAP bacteria represented only 2% of the total bacteria in the NPSG. Despite their lower abundance, they are an active part of the bacterioplankton, with higher growth rates than the average bacteria. This explains the seeming conflict between the reported low AAP abundances (5, 13) and high expression activity of genes encoding anoxygenic photosynthesis (30) in this area. Furthermore, AAP bacteria contributed substantially to the secondary (bacterial) production in the NPSG. In the euphotic zone, there were 3 to 10 million AAP cells per liter being removed at rates of approximately 0.75–0.9 d^−1^. Assuming that an average AAP cell contains 20 fg per cell (31), the AAP community produces 45 to 180 ng of carbon per liter per day. This is approximately 60% of the AAP bacterial production estimated using the same approach for the phosphorus-limited Sargasso Sea (31). Thus, AAP bacteria appear to be an important part of the microbial community in oligotrophic subtropical ocean gyres, regardless of phosphorus or nitrogen limitation.

## MATERIALS AND METHODS

### Sampling

This work was conducted in the NPSG near the Hawaii Ocean Time Series Station ALOHA (22°45′N 158°00′W) aboard the R/V Kilo Moana (cruise KM1013, https://www.bco-dmo.org/dataset/3583) on 13–22 July 2010. During the cruise, 41 individual casts to the euphotic zone were performed using a sampling rosette equipped with Niskin bottles and conductivity, temperature and depth (CTD) sensors.

AAP and total prokaryote abundances in the water column were determined by epifluorescence microscopy as described before (33). Briefly, 5- or 10-mL formaldehyde-fixed water samples were collected onto translucent 0.2-µm polycarbonate filters (Whatman PLC), stained with 4′,6-diamidino-2-phenylindol (DAPI) and counted using an Olympus BX51TF microscope equipped with a monochromatic CCD camera F-ViewII.

### Fluorometry

BChl concentration was estimated from a variable part of BChl fluorescence using an ultra-sensitive infra-red kinetic fluorometer assembled using a standard PSI fluorometer control unit (FL200/PS, Photon Systems Instruments Ltd., Brno, Czechia) and custom-made optics as described earlier (31). To separate Chl *a* and BChl *a* signals, we used the herbicide Diuron to selectively inhibit Photosystem II in phytoplankton as described earlier (38). The 30-µs-long flashes were generated by two units populated with four blue Luxeon diodes (LXHL PB09, 470 nm). The measuring protocol started with an intense multiple-turnover pulse composed of 140 individual flashes (350-µs repetition time, total length 50 ms), which temporarily saturated the RCs to record the maximum fluorescence level. Then, the fluorescence relaxation kinetics was followed by 24 logarithmically spaced flashes during 250 ms after the saturating pulse. The fluorescence signal was averaged over 25 repetitions, with 5 s of dark periods. The electron transfer rate was determined by a single exponential decay fitting of the fluorescence relaxation kinetics following the multiple-turnover pulse (36). The instrument was calibrated using diluted cultures of *Roseobacter* sp. COL2P with known concentrations of BChl *a*. The absolute detection limit was ~0.2 ng of BChl *a* L^−1^ and 1 ng of Chl *a* L^−1^.

### *In situ* BChl *a* decay measurements

BChl measurements were conducted during the daylight hours from 14 to 17 July 2010. Water samples were collected from five consecutive CTD casts while the ship was following the same water mass (drifting at 0.12–0.31 km/h). The first CTD cast was performed at 7:00 a.m. (approximately 1 h after sunrise); the other casts followed approximately every 3 h till sunset. BChl fluorescence measurements were performed on fresh samples within 30 min after CTD recovery. The recorded changes of BChl *a* signal over the course of the day were analyzed mathematically for each depth separately assuming simple exponential decay kinetics: [BChl *a*]_t_ = [BChl *a*]_start_ × exp (*−Dt*), where *t* is the time, *D* is the decay rate constant, [BChl *a*]_t_ is the BChl *a* signal at time *t*, and [BChl *a*]_start_ is the initial BChl *a* signal recorded in the morning (38). The curve fitting was performed using SigmaPlot v.11 (Systat Software Inc., UK).

### On-deck experiments

Seawater for two on-deck experiments was collected from cast #8 at 25-m depth (mixed layer) on 15 July 2010 at 5 a.m. First, a simple dilution experiment was conducted to assess the growth rates of AAP and total bacteria under minimized grazing pressure. We have used a similar methodological approach previously (24, 26, 47). The water was transferred to acid-washed 20-L polycarbonate carboys using acid-washed Tygon tubing. Triplicate carboys were prepared with 2 L of whole seawater and 18 L of seawater gravity filtered through an acid-cleaned 0.2-µm Polycap 36 TC filter (hydrophilic polyethersulfone membrane, Whatman) (1:9 dilution). The carboys were incubated in on-deck flow-through incubators at ambient surface temperature (25.9–26.1°C), covered in foil and two layers of black plastic to maintain darkness. Carboys were sampled at 0, 12, 24, 36, 48, and 72 h. The growth of total and AAP bacteria was monitored using epifluorescence microscopy, and the specific growth rate (µ) was determined by curve fitting, assuming exponential growth kinetics.

Second, a nitrogen enrichment experiment was conducted to establish the nutrient limitation of the AAP growth. Its methodology was described previously (45). Briefly, the collected water was pre-filtered through a 202-µm Nitex® mesh to remove large zooplankton and placed in acid-cleaned (10% HCl) and sample-rinsed 4-L polycarbonate bottles. Based on the typical measurements at Station ALOHA in July [TDN ~ 6 µmol L^−1^, TDP ~250 nmol L^−1^, TDN:TDP ratio ~21 (48)], we prepared three treatments as follows: (i) control (Ctrl, no amendment) (ii); NP32, which was amended with 3 µmol L^−1^ of dissolved inorganic N [as equimolar additions of sodium nitrate (NaNO_3_) and ammonium chloride (NH_4_Cl)] to increase the molar N:P ratio to 32; and (iii) NP50, amended with 8 µmol L^−1^ of dissolved inorganic N to increase the N:P ratio to 50. All bottles were incubated in on-deck blue-shielded Plexiglas (Arkema 2069, 1/4-inch thickness, 50% transmitted light) incubators cooled with surface seawater. Samples for fluorometry and microscopy were collected at the start of the experiment (*T*_*0*_) and after 48, 96, and 144 h of incubation. We prepared 12 replicate bottles for each treatment so that at each time point, we sacrificed triplicate randomized bottles to avoid perturbation and potential contamination of the incubations.
